# Draft genome sequence of *Limosilactobacillus reuteri,* isolated from human breast milk

**DOI:** 10.1128/MRA.01128-22

**Published:** 2023-08-09

**Authors:** Akanksha Tyagi, Su-Jung Yeon, Ramachandran Chelliah, Deog-Hwan Oh

**Affiliations:** 1 Department of Food Science and Biotechnology, College of Agriculture and Life Sciences, Kangwon National University, Chuncheon, South Korea; 2 Kangwon Institute of Inclusive Technology (KIIT), Kangwon National University, Chuncheon, South Korea; 3 Saveetha School of Engineering, SIMATS University, Sriperumbudur, Tamil Nadu, India; University of Maryland School of Medicine, Baltimore, Maryland, USA

**Keywords:** whole genome, gram-positive bacteria

## Abstract

*Limosilactobacillus reuteri* is a lactic acid bacterium with several probiotic properties. Here, we present the draft genome sequence of *L. reuteri* isolated from human breast milk. The average genome size was estimated as 2,087,202 bp, with a guanine-cytosine (GC) content of 51.6%. GC content is the percentage of nitrogenous bases in a DNA or RNA molecule that are either guanine (G) or cytosine (C). De Man, Rogosa and Sharpe, often abbreviated to MRS, is a selective culture medium designed to favor the luxuriant growth of lactobacilli for lab study.

## ANNOUNCEMENT

The present work represents the draft genome sequence of *Limosilactobacillus reuteri* to understand its safety, probiotic characteristics, and technological genomic properties. The present strain was isolated from a 36-year-old woman’s breast milk in India. Fresh breast milk (1 mL) was serially diluted, and 10^9^ dilutions were applied to the MRS media (1). The inoculum was incubated in anaerobic conditions at 37°C for 24 h. Later, 16S rRNA sequencing was performed, and the strain was confirmed as KT000042 *Limosilactobacillus reuteri*.

KT000042 was anaerobically and statically grown in MRS broth for 24 h at 37°C in preparation for long-read sequencing. Genomic DNA (gDNA) was extracted with the Maxwell RSC system from Promega (Promega, Madison, WI, USA), and sheared to 8–13 kb with the Megaruptor 3 system from Diagenode (Megaruptor 3; Diagenode, Denville, NJ, USA); finally, AMPure PB beads were used to remove small fragments (<3 kb) (Pacific Biosciences [PacBio], CA, USA]. The sequencing library was built with the SMRTbell Express template preparation kit v2.0 (PacBio) with 3 µg of gDNA. The Sequel system (PacBio) was used to sequence the library, yielding 72,860 subreads, N_50_ 10,519 bp, with an average length of 9,035 bp. The data were assembled in SMRT Link v10.1.0.119588 (PacBio) using the Microbial Assembly protocol, comprising reads quality control, error correction, adapter filtering, circularity checking, and overlap trimming (2). For short-read sequencing, 100 ng of gDNA was sheared using Adaptive Focused Acoustics technology (Covaris, MA, USA), and a ~350 bp sequencing library was formed using the TruSeq Nano DNA high-throughput library preparation kit (Illumina, San diego, CA, USA). The HiSeq X Ten platform (Illumina) was used for the sequencing, which generated 33,384,528 raw reads and 19,646,034 filtered paired-end reads (90% of bases with a Phred score >30 or higher). The Illumina reads were then mapped to the PacBio assembled genome using BWA-MEMv0.7.17 (3) after adapter and quality trimmed with CANU v1.5 (4). The final error correction was conducted three times with Pilon v1.21 (5) with a default minDepth value of 0.015, resulting in a total of 14 contigs spanning 2,087,202 bp with an average contig length of 682,680 bp, N_50_ of 496,276 bp, 51.6% G = C, and 43.2 Χ total depth (Table 1). The genome annotation by the NCBI Prokaryotic Genome Annotation Pipeline (PGAP) v6.1 (6) and Prokka (v1.13) (7) was used for gene prediction and basic annotation with following options: compliant, rnammer, and addgenes predicting 2,051 protein-coding genes, 59 tRNA genes, 15 rRNA genes, 9 rRNA (5S) genes, 6 16S rRNA genes, and 3 23S rRNA genes. For additional annotation, predicted protein sets were subjected to InterProScan v5.30.69.0 (8) and psi blast v2.4.0 with EggNOG database v4.5 (9). All tools were run with default parameters unless otherwise specified.

**TABLE 1:** Summary of assembly and annotation of draft genome

Names	Contig 1	Contig 2	Contig 3	Contig 4	Contig 5	Contig 6	Contig 7	Contig 8	Contig 9	Contig 10	Contig 11	Contig 12	Contig 13	Contig 14
Feature	Chromosomal	Chromosomal	Chromosomal	Chromosomal	Chromosomal	Chromosomal	Chromosomal	Chromosomal	Chromosomal	Chromosomal	Chromosomal	Chromosomal	Plasmid	Plasmid
Length (bases)	682,680	496,276	240,718	150,217	105,335	93,424	71,775	63,696	57,359	57,148	26,488	19,602	12,854	9,630
GC%	52.3	52.0	51.6	47.5	52.3	53.7	49.7	51.6	52.1	49.2	48.5	53.4	44.0	53.2
CDSs^*a*^	638	501	249	146	106	105	69	60	59	49	21	17	18	13
Depth	40.7	40.5	63.3	46.0	33.8	41.7	51.9	41.3	32.2	49.9	39.5	12.8	6.5	16.1
tRNA	24	2	22	0	1	1	0	2	1	0	6	0	0	0
rRNA	9	0	3	0	0	0	0	0	0	0	3	0	0	0
Total length (bases)	2,087,202													
Total GC%	51.6%													
Total CDS	2,051													
Total depth	43.2													
Coverage % (>=1x)	99.75%													
GenBank accession no.	JAMKCD000000000													
BioProject accession no.	PRJNA834610													
BioSample accession no.	SAMN28052912													
SRA accession no.	SRR21943033													

^*a*^ CDS (coding sequence) is the coding region of a gene.

The present genome was found closer to *L. fermentum* NZ_BJLV00000000.1. In contrast*,* 16S rRNA and our previous research studies (10, 11) confirm it as *L. reuteri* strain. Additionally, literature has evidence that *L. reuteri* is an *L. fermentum* biotype II (12); therefore, the similarity is possible between the strains.

## Data Availability

The draft genome sequence has been deposited in the GenBank database under the accession number JAMKCD000000000, BioProject number PRJNA834610, and BioSample number SAMN28052912 and in the Sequence Read Archive (SRA) database under accession number SRR21943033.
